# Rationale and Design for the BLOCK-SAH Study (Pterygopalatine Fossa Block as an Opioid-Sparing Treatment for Acute Headache in Aneurysmal Subarachnoid Hemorrhage): A Phase II, Multicenter, Randomized, Double-Blinded, Placebo-Controlled Clinical Trial with a Sequential Parallel Comparison Design

**DOI:** 10.1007/s12028-024-02078-z

**Published:** 2024-08-13

**Authors:** Katharina M. Busl, Cameron R. Smith, Andrea B. Troxel, Maurizio Fava, Nicholas Illenberger, Ralisa Pop, Wenqing Yang, Luciola Martins Frota, Hanzhi Gao, Guogen Shan, Brian L. Hoh, Carolina B. Maciel, BLOCK-SAH Investigators

**Affiliations:** 1 Department of Neurology, College of Medicine, University of Florida, McKnight Brain Institute, L3-100, 1149 Newell Drive, Gainesville, FL 32608, USA.; 2 Department of Neurosurgery, College of Medicine, University of Florida, Gainesville, FL, USA.; 3 Department of Anesthesiology, College of Medicine, University of Florida, Gainesville, FL, USA.; 4 Department of Population Health, New York University Grossman School of Medicine, New York, NY, USA.; 5 Department of Psychiatry, Massachusetts General Hospital, Boston, MA, USA.; 6 Department of Biostatistics, University of Florida, Gainesville, FL, USA.; 7 Albany Medical College, Albany, USA.; 8 Emory University, Atlanta, USA.; 9 Johns Hopkins University, Baltimore, USA.; 10 Mayo Clinic Jacksonville, Jacksonville, USA.; 11 Mayo Clinic Rochester, Rochester, USA.; 12 Medical College of Wisconsin, Milwaukee, USA.; 13 Oregon Health and Science University, Portland, USA.; 14 The University of Utah, Salt Lake City, USA.; 15 Thomas Jefferson University, Philadelphia, USA.; 16 University of Florida, Gainesville, USA.; 17 University of Rochester Medical Center, Rochester, USA.; 18 University of Washington, Seattle, USA.; 19 New England Survey Systems, Brookline, USA.; 20 Clinical Coordinating Center at Massachusetts General Hospital, Boston, USA.

**Keywords:** Subarachnoid hemorrhage, Headache, Opioid analgesics, Nerve block, Intensive care

## Abstract

**Background:**

Acute post-subarachnoid hemorrhage (SAH) headaches are common and severe. Management strategies for post-SAH headaches are limited, with heavy reliance on opioids, and pain control is overall poor. Pterygopalatine fossa (PPF) nerve blocks have shown promising results in treatment of acute headache, including our preliminary and published experience with PPF-blocks for refractory post-SAH headache during hospitalization. The BLOCK-SAH trial was designed to assess the efficacy and safety of bilateral PPF-blocks in awake patients with severe headaches from aneurysmal SAH who require opioids for pain control and are able to verbalize pain scores.

**Methods:**

BLOCK-SAH is a phase II, multicenter, randomized, double-blinded, placebo-controlled clinical trial using the sequential parallel comparison design (SPCD), followed by an open-label phase.

**Results:**

Across 12 sites in the United States, 195 eligible study participants will be randomized into three groups to receive bilateral active or placebo PPF-injections for 2 consecutive days with periprocedural monitoring of intracranial arterial mean flow velocities with transcranial Doppler, according to SPCD (group 1: active block followed by placebo; group 2: placebo followed by active block; group 3: placebo followed by placebo). PPF-injections will be delivered under ultrasound guidance and will comprise 5-mL injectates of 20 mg of ropivacaine plus 4 mg of dexamethasone (active PPF-block) or saline solution (placebo PPF-injection).

**Conclusions:**

The trial has a primary efficacy end point (oral morphine equivalent/day use within 24 h after each PPF-injection), a primary safety end point (incidence of radiographic vasospasm at 48 h from first PPF-injection), and a primary tolerability end point (rate of acceptance of second PPF-injection following the first PPF-injection). BLOCK-SAH will inform the design of a phase III trial to establish the efficacy of PPF-block, accounting for different headache phenotypes.

## Introduction

### Study Rationale

Post-subarachnoid hemorrhage (SAH) headaches are a major challenge for patients to endure and for clinicians to treat [1]. As many as 90% of patients with SAH experience severe headaches (i.e., scored ≥ 7 on a 0–10 numeric rating scale) [2], and more than half report a maximal pain score of 10 at some point during their hospitalization [2]. On average, patients with SAH have severe headaches for ≥ 7 days [3, 4]. Control of acute post-SAH headaches is overall poor, with persistence of severe pain despite a combination of analgesics [2, 3]. Opioids, either alone or in combination, are perceived as the most effective available treatment for post-SAH headache [1] and remain the guideline-recommended mainstay of acute therapy for severe pain [5]. In addition to their addiction-bearing potential, opioids come with numerous side effects, including altered consciousness and depressed respiratory drive, constipation, and hypotension [6]. Opioid-sparing analgesic strategies for post-SAH headache with gabapentin, pregabalin, corticosteroids, and magnesium provide only modest pain relief and are hampered by their own set of side effects [7–10]. Moreover, escalating opioid doses are often required despite opioid-sparing analgesics [3, 11, 12]. Importantly, uncontrolled pain during hospitalization is associated with continued opioid use after discharge [12, 13].

The impact of post-SAH headache is daunting; it is the fourth most common cause of 30-day and 90-day readmission to the hospital [14] and significantly reduces quality of life [15, 16]. Although pain experiences differ across individuals [17], the common experience among survivors is that current therapies provide inadequate pain relief, and effective opioid-sparing alternatives for post-SAH headache are urgently needed.

Peripheral nerve blocks, as a component of multimodal analgesia or replacement for systemic analgesics [18], are effective for the treatment of headaches [19–21]. Pterygopalatine fossa (PPF) nerve blocks target the sphenopalatine ganglion (SPG)—an autonomic ganglion containing parasympathetic vasomotor fibers responsible for the vasodilatory trigemino-autonomic pain reflex—and the maxillary division of the trigeminal nerve (V_2_). It functions as a switching nucleus for autonomic fibers [22] and is believed to play a key role in headache generation [23]. Mechanisms of headache generation following SAH are not fully understood and are likely variable, including meningeal irritation by blood products [24], release of inflammatory cytokines [25], vasomotor instability, and central sensitization by glutamatergic N-methyl-D-aspartate receptors [26]. Further, nociceptive input from trigeminal and trigeminovascular fibers that innervate head and neck tissues are activated following SAH [24]; these also innervate extracranial and meningeal vessels as well as proximal intracranial arteries in the Circle of Willis. Analgesic efficacy of SPG- and PPF-blocks has been demonstrated in the care of patients requiring midface or pharyngeal surgeries [27, 28], and the body of promising data in acute and chronic headache is growing, indicating pain control for hours to days with a single block and for up to 6 months with repeated blocks [29–32].

Our preliminary experience with PPF-blocks for post-SAH headache [33, 34], offered to patients for refractory headache during hospitalization for SAH, indicates that PPF-blocks may be a promising, opioid-sparing, and safe treatment option for patients with SAH.

### Objective

The objective of the BLOCK-SAH trial is to assess the efficacy and safety of bilateral PPF-blocks with ropivacaine and dexamethasone when administered early during hospitalization for acute aneurysmal SAH in patients with post-SAH headaches.

### Hypothesis

Our central hypothesis is that PPF-blocks mitigate opioid requirements, improve pain control for acute post-SAH headaches, and are a safe and well-tolerated alternative to existing standard pain management strategies.

## BLOCK-SAH Study Design

The protocol was designed following the SPIRIT 2013 (Standard Protocol Items: Recommendations for Interventional Trials) statement [35]. The BLOCK-SAH trial is conducted in accordance with the Declaration of Helsinki and the US Code of Federal Regulations applicable to clinical studies. The investigators further follow the International Conference on Harmonization Good Clinical Practices Guidelines. The study protocol has been approved by the trial’s central institutional review board (IRB) and will be approved by each local IRB prior to any patient-facing study-related procedures.

### Synopsis

**Fig. 1:**
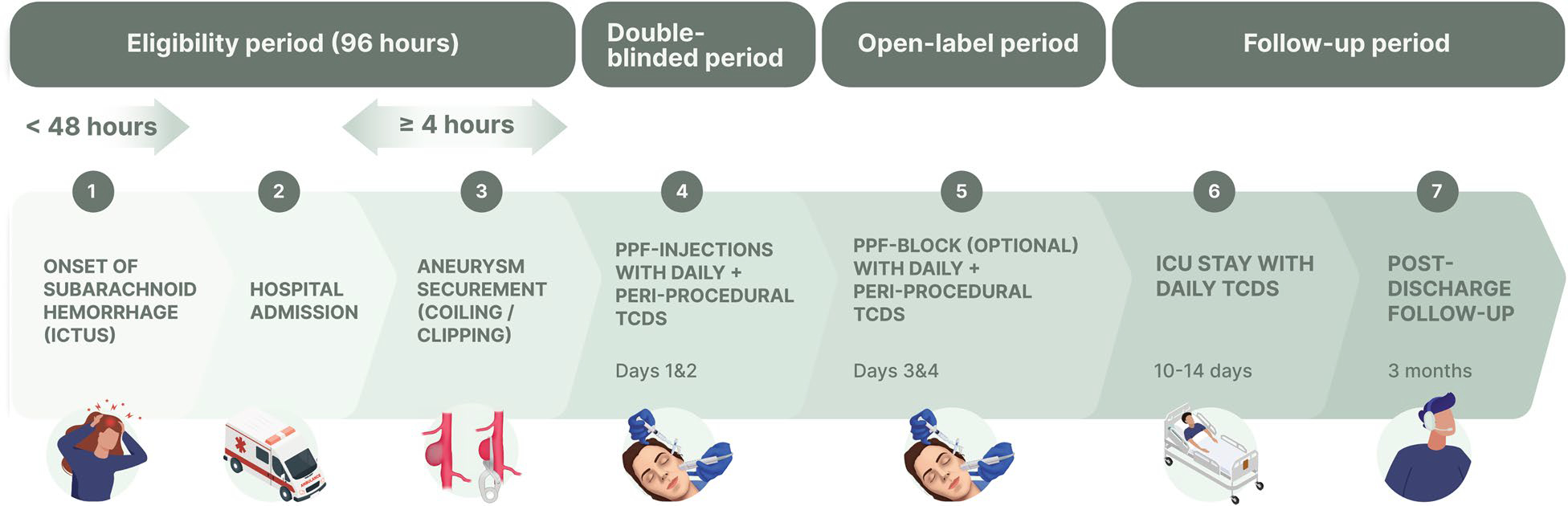
Study timeline depicting the participant-facing events of BLOCK-SAH. ICU intensive care unit, TCD transcranial Doppler

BLOCK-SAH is a phase II, multicenter, randomized, double-blinded, placebo-controlled clinical trial of bilateral PPF-injections (active PPF-block with a 5-mL solution of 20 mg of ropivacaine plus 4 mg of dexamethasone vs. placebo with 5 mL of saline) for headache in awake survivors of aneurysmal SAH, with monitoring of intracranial arterial mean flow velocities with transcranial Doppler (TCD) within 3 h before and after PPF-injections. BLOCK-SAH is expected to have a total study duration of 4 years. The subject-facing part of the study occurs in four periods: eligibility and screening, double-blinded, open-label, and follow-up (see Fig. 1). The follow-up observation period involves the remaining hospitalization through discharge and a 3-month follow-up visit.

### Eligibility Criteria

Adult patients hospitalized with a primary diagnosis of spontaneous SAH will be screened for potential eligibility against the study protocol inclusion and exclusion criteria. A total of 195 hospitalized awake patients with the primary diagnosis of aneurysmal SAH will be enrolled. Participants must meet all of the inclusion criteria and none of the exclusion criteria to be eligible for participation in the study (see Table 1). Eligibility criteria were established with an emphasis on maximizing safety. As such, we are including only aneurysmal SAH after complete securement of culprit aneurysm (i.e., predictably reduced risk of rebleeding after documented aneurysm securement) within strict eligibility time windows (to avoid peak vasospasm period, the first set of PPF-injections must be completed within 96 h of ictus); we are excluding patients with vasospasm or flow-limiting narrowing of intracerebral vasculature and patients with coagulopathies or requiring systemic anticoagulation or expanded antiplatelet therapy. To assess the hypothesized opioid-sparing effect, a minimum requirement of opioid consumption is warranted pre-enrollment. The chosen cutoff at 15 mg of oral morphine equivalent (OME)/day for headache analgesia over a 24-h period during the eligibility period is conservative based on average reported opioid requirements in modern cohorts [3, 11, 12]. Patients with premorbid chronic use of opioids or barbiturates and patients with substance use disorders are ineligible.

### Randomization

Participants will be randomized to one of the three study groups in a 1:1:1 ratio, in accordance with the sequential parallel comparison design (SPCD) described in the Study methodology section, and stratified by site and treatment modality (craniotomy with clipping vs. endovascular aneurysm obliteration). To prevent randomization imbalances by race and sex, we use a covariate adaptive randomization method [36].

### Intervention

**Fig. 2:**
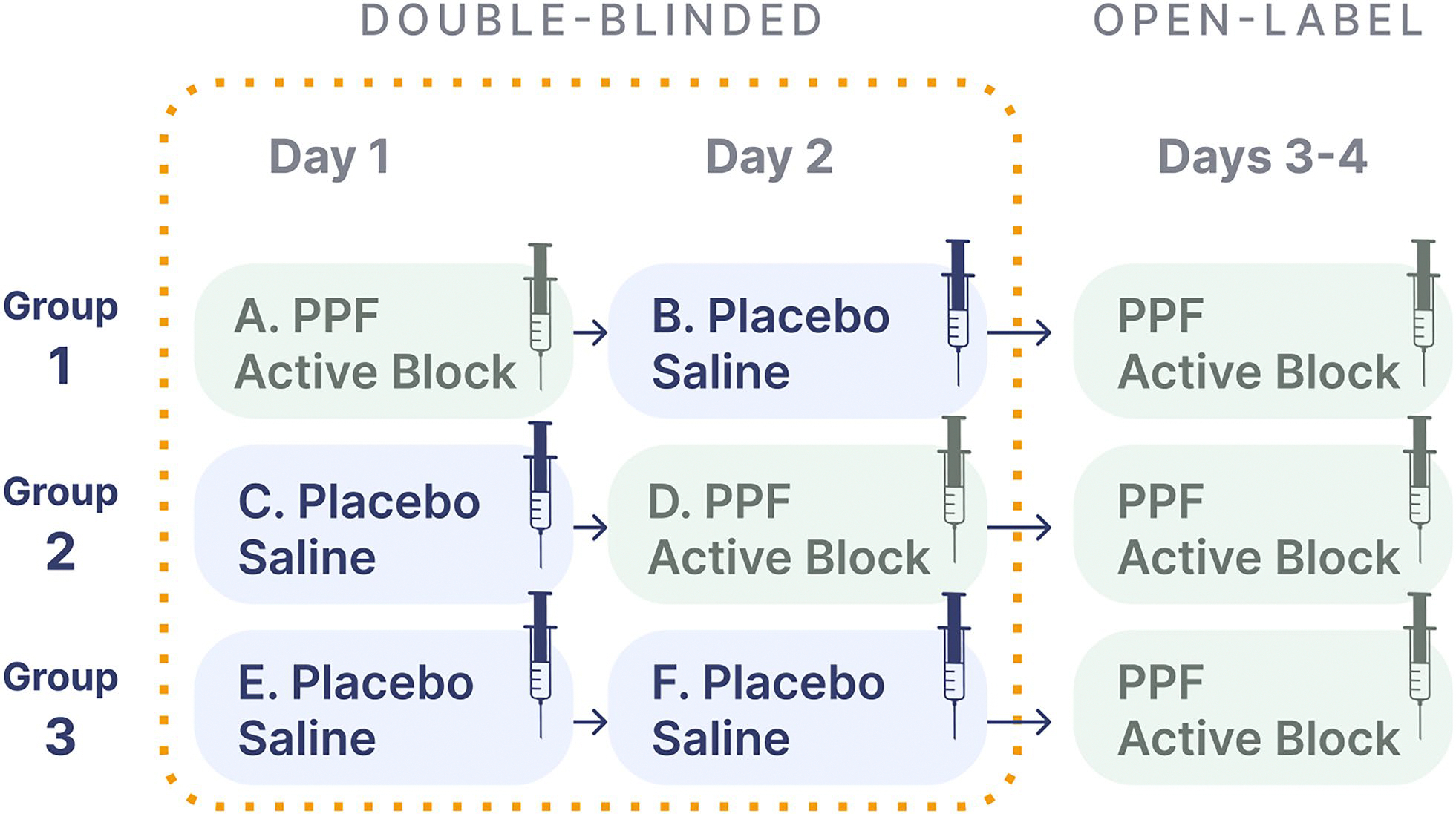
Study design: Sequential Parallel Comparison Design. PPF: pterygopalatine fossa

**Fig. 3:**
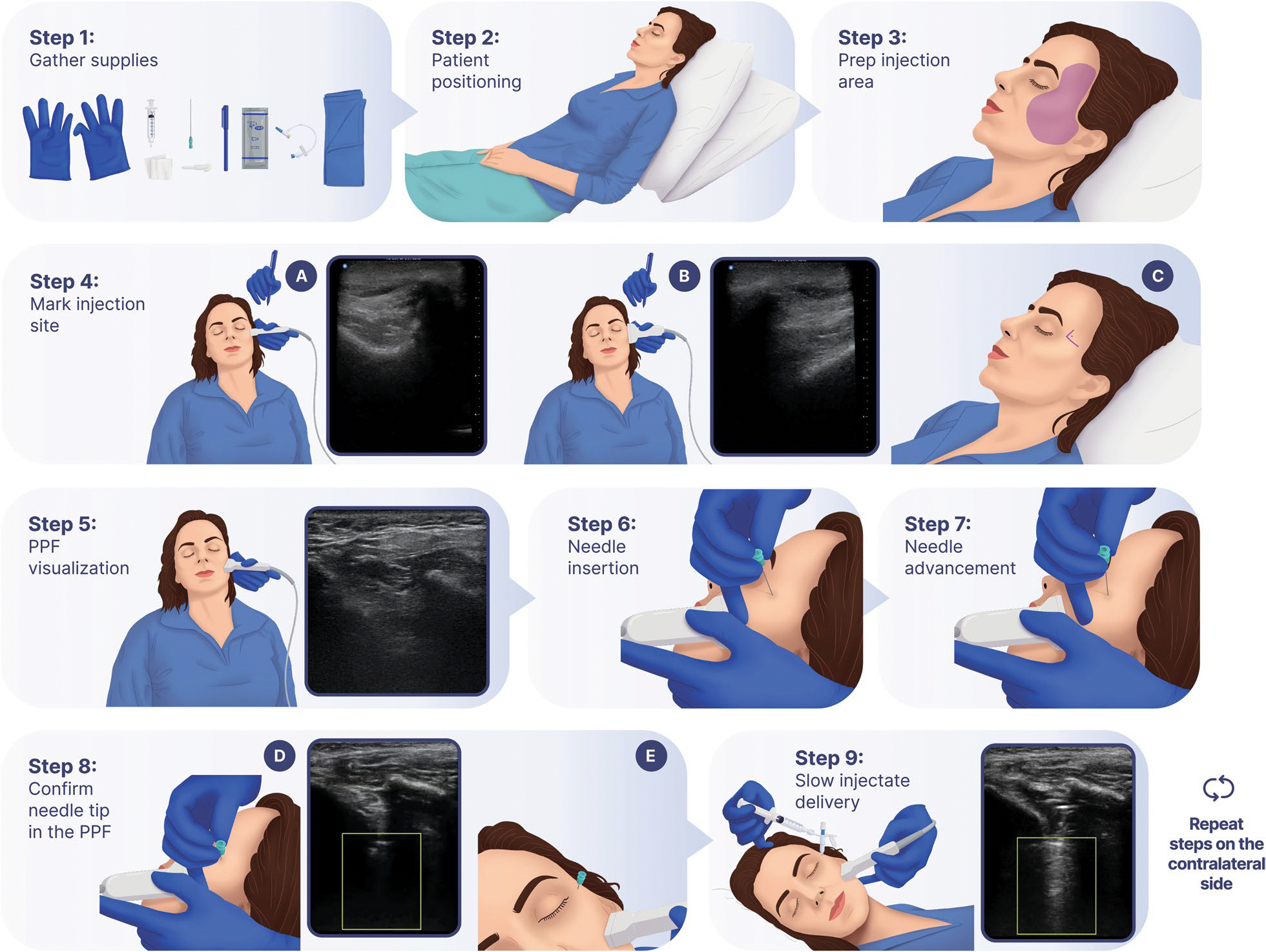
Intervention: pterygopalatine fossa- (PPF-) injection. Steps 1–9 outline how the intervention is performed on each side. Step 4 A: ultrasound image depicting the posterior orbital rim. Step 4 B: ultrasound image depicting the superior edge of the zygomatic arch. Step 5: ultrasound image depicting the space between maxilla and coronoid process of the mandible. Step 8 D: ultrasound image depicting the needle tip located in the PPF. Step 9: ultrasound clip/image depicting injectate spread in the PPF

Each participant will receive bilateral PPF-injections once daily for 2 consecutive days (see Fig. 2). The PPF-injection is conducted at the bedside via the suprazygomatic approach under ultrasound guidance [33]. The injectate consists of a total of 5 mL of either 4 mL of 0.5% ropivacaine combined with 1 mL of dexamethasone (active block) or 5 mL of saline (placebo injection) and is prepared by the local investigational pharmacy and dispensed to the procedural study team in a blinded fashion. The steps of the sterile procedure as conducted in BLOCK-SAH are outlined in Fig. 3. A standardized set of ultrasound images and cine clips (i.e., sonographic view of the PPF prior to needle insertion, appropriate needle position in the PPF, injectate delivery, and muscle displacement by injectate after delivery) will be saved for each injection to be transmitted for review by the procedural competency committee.

### Procedural Rigor

All site proceduralists undergo a rigorous, standardized procedural training, including review of anatomy and procedural technique and hands-on learning via a high-fidelity simulator, as well as a structured assessment of procedural competence. To ensure highest level procedural compliance during individual enrollments, BLOCK-SAH instituted a procedural competency committee composed of five independent experts in conduction of PPF-blocks and led by procedural multi-principal investigator Dr. Smith. This committee will assess each PPF-injection conducted at participating sites during active enrollments within 12 h of the injection. The site investigator team electronically submits the ultrasound still images and cine clips obtained during each PPF-injection. Placement of PPF-injection will be graded independently by two members of the procedural competency committee in addition to the procedural multi-principal investigator and graded using Delphi consensus methodology as adequate, inadequate, or indeterminate. A remediation plan will be implemented prior to subsequent injections if necessary.

### Monitoring and Assessments

#### Vasospasm Surveillance

Large-vessel vasospasm occurs in approximately 45% of patients with SAH [37–39], with < 30% facing severe vasospasm [40]. The American Heart Association guidelines recommend serial TCD monitoring of mean velocities in the Circle of Willis for early vasospasm detection [41]. All consortium sites are conducting daily TCD monitoring for patients admitted with aneurysmal SAH. In addition, periprocedural TCDs within 3 h before and after PPF-injections will allow detection of transient sonographic vasospasm (i.e., mean flow velocities > 120 cm/s [37, 42]) that could be missed on daily monitoring.

#### Periprocedural Monitoring of Cerebral Blood Flow

Both preganglionic and postganglionic parasympathetic and sympathetic fibers cross the PPF. Vasodilation is likely mediated by selective parasympathetic fibers connected to vascular beds of the cerebral hemisphere [43–45], but changes in vessel caliber may also occur because of an indirect effect through sympathetic fibers innervating blood vessels and traversing the PPF [46]. Cerebrovascular modulation is complex [45]: The relationship between SPG stimulation with changes in cerebral blood flow is largely transient and inconsistent across studies [43, 44, 47], and a potential effect of PPF-blocks on vasomotor reactivity in the setting of SAH remains largely unknown. To date, there are no definitive reports of vasospasm following SPG-block, irrespective of the extent of the block (i.e., if isolated to SPG or all structures in the PPF), including in our own preliminary data [33, 34]. On the other hand, patients with SAH with higher pain scores are also more likely to experience early increases in cerebral blood flow velocity [48], suggesting a higher risk of vasospasm in the setting of poorly controlled pain [49]. The theoretical concern for vasospasm motivates the primary BLOCK-SAH safety outcome—the incidence of radiographic vasospasm at 48 h from first PPF-injection—given the potential for morbidity associated with vasospasm and delayed cerebral ischemia in SAH [37]. Hence, we will monitor cerebrovascular flow velocities with TCD within 3 h before and within 3 h after every PPF-injection, regardless of study phase (i.e., in both double-blinded and open-label phases). If TCDs cannot be obtained because of insufficient bone windows, a scheduled computed tomography angiography will be obtained at two prespecified time points. Study participants with radiographic severe vasospasm (mean velocities > 200 cm/s [40, 50, 51] or determined by angiography) or deemed to have clinically significant vasospasm by the clinical team will be ineligible for subsequent PPF-injections.

#### Clinical Monitoring

Pain is assessed hourly as a standard of care in participating sites during neurochecks [52] with the 0–10 pain numeric rating scale [53]. Delirium will be evaluated with the Confusion Assessment Method [54] every 12 h, as level of pain control and opioid use are associated with delirium in the critically ill population [55]. Need for and degree of cerebrospinal fluid diversion and intracranial pressure will be captured, when applicable.

#### Diagnostic Assessments

Questionnaires to capture the perceived impact of pain intensity, maladaptive coping mechanisms, and highly prevalent comorbid conditions (e.g., tobacco dependence and anxiety) that may mediate disparities in outcomes [56–60], pain experiences [61, 62], and treatment responses will be applied according to the prespecified schedule of events during visits with the participants.

### Study End Points

The primary efficacy end point is the pro re nata (i.e., “as needed”) OME/day use within 24 h after each PPF-injection spanning the 48 h of the double-blinded treatment period. The primary safety end point is the incidence of radiographic vasospasm at 48 h from first PPF-injection, and the primary tolerability end point is the rate of acceptance of a second PPF-injection at 24 h following the first PPF-injection. Secondary end points include mean hourly pain scores within 24 h after each PPF-injection, rates of adverse events related to PPF-injection, incidence of radiographic vasospasm at 24 h after first active PPF-injection, incidence and magnitude of radiographic vasospasm from the end of the double-blinded treatment period until the end of the intensive care unit stay, and rates of agreement to active PPF-block (during the open-label period) and multiple PPF-injections. Exploratory aims include assessment of headache burden in the medium term (including assessment of opioid use throughout hospitalization and during the follow-up period) and differences in the pain experience, efficacy, and tolerability of PPF-block by sociodemographic and clinical characteristics.

### Study Methodology

The trial is designed according to SPCD. This design involves three intervention arms: (1) active (stage 1), placebo (stage 2); (2) placebo (stage 1), active (stage 2); and) (3) placebo (stage 1), placebo (stage 2) (see Fig. 2 SPCD). We chose this design over a conventional two-arm comparison design because of the possibility, if not likelihood, of a large expected placebo effect. The placebo effect in pain interventions varies widely, and placebo analgesia can be significant [63, 64]. SPCD leverages the partial enrichment in placebo nonresponders with expected reduction in placebo response in the trial’s stage 2, resulting in increased efficiency (as more data are generated from a given sample size). This mitigates a potentially high response to placebo [65], which is important in the setting of a placebo intervention involving a procedure (injection of saline) and patient-reported outcomes—all factors associated with higher placebo effect [66, 67]. All enrolled participants still meeting safety criteria will be offered the opportunity to request a guaranteed active PPF-block in the open-label period following the double-blinded phase.

### Blinding

Participant allocation to one of the three intervention groups during the double-blinded period occurs quadruple-masked (participant, care team, investigator/study team, and outcome assessor).

#### Standardized Care

All participating sites use a standardized pain regimen, including medications that must be administered or offered and medications that are not allowed during prespecified time points (Table 2). Eligible patients (during the eligibility period) and enrolled study participants (during the double-blinded phase) must receive scheduled acetaminophen (maximum of 4 g/day or dose-adjusted according to hepatic function) regardless of pain intensity and must have access to opioids as needed for intolerable headache (according to patient response to the hourly pain assessment question: “Is the headache tolerable?”). Barbiturate analgesics or glucocorticosteroids are not permitted for headache management during the eligibility period and double-blinded phase but are allowed subsequently. Multimodal analgesia with gabapentin, pregabalin, magnesium, duloxetine, venlafaxine, and cyclobenzaprine is permissible either scheduled or as needed regardless of the phase of the study.

#### Follow-Up

One follow-up visit, either in-person or remotely, will be conducted at 3 months post discharge, during which all enrolled study participants will be assessed for continued opioid use and occurrence and severity of persistent headache through questionnaires.

#### Statistical Analysis

The sample size of 195 provides sufficient power for placebo response rates up to 30% and includes 5% attrition after the first injection for study participants who decline a second injection. The sample size was determined to have 80% statistical power to detect the active-placebo difference of a small to medium Cohen’s effect size with *d* = 0.35 in stage 1 and *d* = 0.39 in stage 2 (using a two-sided type 1 error of 0.05). There is clear evidence that stage 2 of SPCD is associated with a lower placebo/sham response and therefore a larger effect size [68]. The statistical analysis plan includes contingency plans for adjustment of sample size to maintain 80% power using SPCD as well as thresholds for changing study conduct and analyses to a simple parallel design in case of higher attrition rates. Regarding the primary safety outcome, the target sample size of 195 provides 80% power to detect at least a 25-percentage-point difference in the occurrence of radiographic vasospasm at 48 h after the first injection between study participants receiving an active PPF-block and those receiving a placebo PPF-injection.

Analyses for all study outcomes will be conducted on the intention-to-treat sample. A single interim safety analysis will be performed when 50% of patients (*n* = 98) have been observed through hospital discharge. There will be no planned interim analysis for the primary efficacy outcome.

All data will be descriptively summarized. Continuous data will be characterized using means, standard deviations, medians, interquartile ranges, minimums, and maximums. Categorical data will be described using counts and percentages across categories. All study hypotheses will be tested using two-sided tests with a significance level of 0.05. No multiple testing adjustment will be applied because each one of them is of unique and specific interest, and this is a proof-of-concept trial. Our planned exploratory analysis considers sex and race and ethnicity when assessing the effect and tolerability of PPF-blocks. All analyses will be conducted using the latest version of the software R.

#### Safety Monitoring

Safety monitoring will be conducted in accordance with the International Conference on Harmonization Good Clinical Practices Guidelines. The study will be monitored by an Independent Medical Safety Monitor (IMSM), the central IRB (Advarra), local IRBs, and a Data Safety Monitoring Board (DSMB) appointed by the National Institutes of Health. The appointed IMSM is an expert in the treatment of patients with aneurysmal SAH and will review all serious adverse events as well as every occurrence of vasospasm to adjudicate its relationship to the injection. DSMB meetings will be held every 6 months, with an option to increase the meeting frequency if needed.

#### Sites and Administration

BLOCK-SAH is led by the investigator team at the University of Florida, in collaboration with the Clinical Coordinating Center (Clinical Trials Network and Institute, Massachusetts General Hospital), and the Data Coordinating Center (New York University Grossman School of Medicine). Participating consortium sites are 12 academic referral centers in the United States with dedicated neurointensive care units and high annual case volumes (i.e., ideally, *n* ≥ 35) of aneurysmal SAH. The trial is registered on ClinicalTrials.gov as NCT06008795. Public or scientific inquiries may be directed to the lead principal investigator, Dr. Katharina M. Busl. Substantive protocol modifications are subject to review by the National Institutes of Health, the DSMB, and the central IRB.

## Discussion

We designed BLOCK-SAH to pioneer the study of nerve blocks as an opioid-sparing therapeutic strategy for severe headaches in the neurocritical care setting. If our central hypothesis is correct, that is, PPF-blocks provide rapid headache relief while reducing opioid requirements and are safe and well-tolerated by patients with SAH, BLOCK-SAH has the potential to establish an opioid-sparing approach that could also be explored in other forms of acute brain injuries associated with headache, such as intraparenchymal hemorrhage [69], traumatic brain injury, and acute ischemic stroke [70]. Furthermore, our initial effort to identify post-SAH headache phenotypes is essential to learn about effect modifiers so that appropriate conclusions about efficacy and tolerability can be made. Studying the potential impact of sociodemographic factors on differential response to pain and its treatment addresses a disparity gap in general critical care [71] and SAH [72], a disease that disproportionally affects women and Hispanic and Black communities [73, 74]. The results of this study will inform a phase III trial to establish the efficacy of PPF-block, accounting for different post-SAH headache phenotypes and disparities in pain experiences.

## Supplementary Material

Supplementary file 1

## Figures and Tables

**Table 1:** Eligibility Criteria for BLOCK-SAH

Inclusion criteria	Exclusion criteria

Regulatory Provision of signed informed consent Willingness to comply with all study procedures	Uncorrected coagulopathy a. Platelet count < 50,000/μL, INR > 1.7 b. Requiring systemic anticoagulation or antiplatelets (except for aspirin monotherapy)
Age and Sex 18–85 years, male or female	SAH-specific a. Head trauma or infection (e.g., mycotic aneurysm) as etiology of SAH b. Inability to successfully treat culprit vascular lesion c. Diffuse vasospasm on initial diagnostic CTA or DSA
SAH-specific a. Spontaneous, non-traumatic, SAH admitted within 48 h of ictus hemorrhage b. Culprit aneurysm identified c. Modified Fisher Grade 1–4 on admission imaging d. Hunt and Hess 1–3 or WFNS grade 1–4* e. GCS verbal subscore ≥ 4	Premorbid conditions a. Preexisting condition confounding neurologic assessment or precluding accurate outcomes assessment b. Preexisting diffuse flow-limiting narrowing of arteries in the Circle of Willis, regardless of etiology (e.g., atherosclerosis, vasculitis, Moya-Moya syndrome) c. Prior use of opioid or barbiturate analgesics for at least 2/3 of days in previous month d. Diagnosis of substance use disorder in the previous year e. Infected or wounded skin, at the site of puncture for PPF-injection
Stabilization period a. Minimum of 4 h from clipping or coiling procedure b. Successful treatment of culprit vascular lesion (i.e., occlusion of ≥ 90%)	Standard pain regimen conditions a. Absolute contraindication for acetaminophen b. Hepatic enzymes (i.e., AST or ALT) > 3 × upper limit level c. Allergy or intolerance to ropivacaine, dexamethasone or standard pain regimen
Headache-specific a. Able to verbalize pain intensity scores b. Requiring ≥ 15 mg OME prn for headache analgesia over a 24 h-period during eligibility period	Others a. Participation in a concurrent investigational/interventional study b. Positive pregnancy test/known to be pregnant; vulnerable populations c. Unable to receive PPF-injection within 96 h of ictus hemorrhage

AST, Aspartate aminotransferase; ALT, Alanine transaminase; CTA, Computed tomography angiography; DSA, Digital subtraction angiography; GCS, Glasgow Coma Scale; INR, International normalized ratio; OME, Oral morphine equivalent; SAH, Subarachnoid hemorrhage; WFNS, World Federation of Neurological Surgeons;

* , must also fulfill GCS verbal subscore ≥ 4

**Table 2:** Standardized headache management and concomitant medications

Medication	Eligibility Period & Double - blinded Period		Open-label Period		After Open-label Periods	
	Standing	prn	Standing	prn	Standing	prn
Acetaminophen (APAP)	!!!	✓	✓	✓	✓	✓
Opioids*	✗	!!!	✓	✓	✓	✓
NSAIDS	✓	✓	✓	✓	✓	✓
Gabapentin	✓	✓	✓	✓	✓	✓
Pregabalin	✓	✓	✓	✓	✓	✓
Magnesium	✓	✓	✓	✓	✓	✓
Duloxetine	✓	✓	✓	✓	✓	✓
Venlafaxine	✓	✓	✓	✓	✓	✓
Cyclobenzaprine	✓	✓	✓	✓	✓	✓
Barbiturates	✗	✗	✓	✓	✓	✓
Glucocorticosteroids	✗	✗	✓	✓	✓	✓
Tramadol	✗	✗	✗	✗	✗^#^	✗^#^
PCA pump	✗	✗	✗	✗	✗^#^	✗^#^
Other craniofacial nerve blocks	✗	✗	✗	✗	✓	✓
Botulinum toxin injections	✗	✗	✗	✗	✓	✓

!!! Mandatory; ✓ Permitted; ✗ Not allowed;

* Opioids: buprenorphine, codeine, fentanyl, hydrocodone, hydromorphone, oxycodone, meperidine, methadone, morphine, oxymorphone and tapentadol can be either oral, enteral, or IV;

# Not allowed during hospitalization

NSAIDS, Non-steroidal anti-inflammatory drugs”; PCA, Patient-controlled analgesia; prn- “as needed
