# Supplementary material for: Rationale and Design for the BLOCK-SAH Study (Pterygopalatine Fossa Block as an Opioid-Sparing Treatment for Acute Headache in Aneurysmal Subarachnoid Hemorrhage): A Phase II, Multicenter, Randomized, Double-Blinded, Placebo-Controlled Clinical Trial with a Sequential Parallel Comparison Design
Source: Neurocrit Care. Author manuscript; Available in PMC 2025 Feb 11. (PMC11810580; doi:10.1007/s12028-024-02078-z)
Supplement: Supplementary file 1 [file NIHMS2040191-supplement-Supplementary_file_1.docx]

**Supplementary File 1: BLOCK-SAH Sites and Investigators**

**Albany Medical College:** Alan Boulos, Andras Laufer, Charles E. Argoff, Christopher Figueroa, Erin Barnes, James Lee, Mahtab Sheikh, Nibras Bughrara, Panayiotis Varelas, Toni Schaeffer

**Emory University:** Christine Spainhour, Ofer Sadan, Owen Samuels, Tommy T. Thomas, Yawar Qadri

**Johns Hopkins University:** Eusebia Calvillo, Jose Ignacio Suarez, Kate Rosenblatt, Tina Tuong-Vi Le Doshi

**Mayo Clinic Jacksonville:** Amber Patchell, Candace Hendricks, Christopher Kramer, Elird Bojaxhi, Ewa Szymkiewicz, Ferenc Rabai, Jeffrey Peel, Lauren Ng, Megan Gauthier, Miriam Anacker, Sindhuja Nimma, W. Christopher Fox, William David Freeman

**Mayo Clinic Rochester:** Alejandro Rabinstein, Amy Headlee, Bridget Neja, Carey Huebert, Chyann Moore, Ethan R. Schlecht, Jane Sultze, Matthew Pingree, Muhib Khan, Narayan Kissoon, Peter Reuter

**Medical College of Wisconsin:** Ali Daha, Anna Curtis, Devin Gillespie, Gregory Rozansky, Gwynne Kirchen, Jacob Labinski, James LaTourette, Jamie Jasti, Jennifer Hernandez-Meier, Linda Mattrisch, Omar Dyara, Oscar Jim Michael Coppes, Sarah Abdallah, Sarah Endrizzi, Tom P. Aufderheide, Vladimir Suric

**Oregon Health and Science University:** Elena Spontak, Ines P. Koerner, Kimberly M. Mauer, Sarah Feller

**The University of Utah:** Connie Chung, Kelsey Dalton, Kevin C. Brennan, Kinga Aitken, Nabeel Chauhan

**Thomas Jefferson University:** Jaime Baratta, Michael Reid Gooch, Nabeel Herial, Nadirah Jones, Pascal Jabbour, Robert Rosenwasser, Stavropoula Tjoumakaris, Wendell Gaskins

**University of Florida**: Adam Crisologo, Ali Mustafa, Amanda Dyer, Amy Gunnett, Andrey Suprun, Anum Khaliq, Bakhtawar Ahmad, Barys Ihnatsenka, Beulah Augustin, Brandon Lucke-Wold, Bronson Crawford, Christopher Robinson, Daniela Pomar-Forero, Federico Jimenez Ruiz, Hector David Meza Comparan, Isaac Luria, Ivan Rocha Ferreira Da Silva, John Bruno, Joshua Wais, Juan Acosta, Kevin Priddy, Laura Glicksman, Linda Le-Wendling, Magali Jorand-Fletcher, Matthew Koch, Matthew Mallard, Melissa Johnson, Michael Anthony Pizzi, Nicholas Nelson, Nohra El Chalouhi, Olga Nin, Patrick Tighe, Pouya Ameli, Richa Wardhan, Sebastián Gatica-Moris, Shilpa Haldal, Soleil Schutte, Svetlana Chembrovich, Thiago Santos Carneiro, Yury Zasimovich

**University of Rochester Medical Center:** Antonia Heininger, Clifton Houk, Derek George, Imad Khan, Mark Williams, Matthew Bender, Pablo Valdes Barrera, Steven Soler, Tarun Bhalla, Thomas Mattingly, Tilor Hallquist

**University of Washington:** Abhijit V. Lele, Adrienne James, Do Lim, Michael Levitt, Michele Curatolo, Sarah Wahlster

**New England Survey Systems:** Angela DeLuca, Charlie Biluck, Daniia Newman, John Roberge, Joseph Miller, Justin Roberge, Mercedes Helm, Michael Stanley, Zoe Belge 

**Clinical Coordinating Center at Massachusetts General Hospital:** Carissa Lin, James P. Rathmell, Martina Flynn, Robert Randolph Edwards, Yurerkis Montas
